# The Impact of First UK-Wide Lockdown (March–June 2020) on Sexual Behaviors in Men and Gender Diverse People Who Have Sex with Men During the COVID-19 Pandemic: A Cross-Sectional Survey

**DOI:** 10.1007/s10508-022-02458-6

**Published:** 2022-11-07

**Authors:** Tom Nadarzynski, Will Nutland, Phil Samba, Jake Bayley, T. Charles Witzel

**Affiliations:** 1 School of Social Sciences, University of Westminster, Room 6.101, 115 New Cavendish Street, London, W1W 6UW UK; 2 Department of Public Health, Environments and Society, London School of Hygiene and Tropical Medicine, London, UK; 3 The Love Tank CIC, London, UK; 4 Sexual Health and HIV Department, Barts NHS Trust, London, UK

**Keywords:** Sexual behaviors, COVID-19, SARS-CoV-2, Sexual minority, Gender minority, Transgender

## Abstract

The global COVID-19 pandemic resulted in strict social distancing measures restricting close physical contact. Men (cis and trans) and other gender diverse people who have sex with men (MGDSM) are at higher risk of sexually transmitted infections (STIs) and may have experienced changes in sexual behavior during government restrictions on social and sexual contact. We aimed to examine self-reported sexual behavior of MGDSM during the first UK-wide lockdown to identify the characteristics of the individuals who might most require sexual health promotion and clinical support. In April–May 2020, we conducted an online survey of MGDSM, promoted on social media and Grindr. Our exploratory approach used descriptive analysis to identify self-reported changes in sexual behavior and performed regression analyses to identify correlates of casual sex during the lockdown. A total of 1429 respondents completed the survey: mean age 36 years, 84% White, 97% male or trans male, 98% assigned male sex at birth, 2% female or non-binary, 65% degree educated or higher. During the lockdown, 76% reported not having any casual sex partners. While the majority reported reduced casual sex, 3% reported an increase in casual sex with one person and 2% with three or more people (group sex). About 12% of the sample engaged in casual sex with only one person and 5% with four or more sexual partners during the lockdown. Reporting casual sex during lockdown was associated with: lower level of education OR = 2.37 [95% CI 1.40–4.01]; identifying as a member of an ethnic minority OR = 2.27[1.40–3.53]; daily usage of sexual networking apps OR = 2.24[1.54–3.25]; being less anxious about contracting SARS-CoV-2 through sex OR = 1.66[1.12–2.44]; using PrEP before lockdown OR = 1.75[1.20–2.56]; continuing to use PrEP OR = 2.79[1.76–4.57]; and testing for STIs during lockdown OR = 2.65[1.76–3.99]. A quarter of respondents remained sexually active with casual partners, indicating a need to provide STI screening services and health promotion targeted to groups most likely to have need over this period. Future research is required to better understand how to support sexual and gender minorities to manage sexual risk in the context of pandemic public health initiatives.

**Supplementary Information:**

The online version contains supplementary material available at 10.1007/s10508-022-02458-6.

## Introduction

Severe acute respiratory syndrome coronavirus 2 (SARS-CoV-2) is a novel coronavirus that causes coronavirus disease 2019 (COVID-19) (Lai et al., 2020). SARS-CoV-2 is a respiratory virus transmitted primarily through aerosols, making it highly contagious, with a relatively high proportion of asymptomatic cases (Long et al., 2020). SAR-CoV-2 first emerged in Wuhan, China, in December 2019. In March 2020, the World Health Organization declared a global pandemic and the UK government introduced strict restrictions on human mobility and socializing recommending against non-essential travel and enforcing social distancing (Cucinotta & Vanelli, 2020). Between 23 March and 4 July 2020, the first nationwide UK lockdown was put into place to reduce excess hospitalization of patients due to COVID-19 and associated mortality (Hadjidemetriou et al., 2020).

There is strong evidence that these measures have had impact on sexual behaviors worldwide. A recent scoping review found reductions in sexual activity during social distancing mandates in Brazil, the USA and Wales, and a decline in sexual well-being in Poland and the USA (Bolarinwa et al., 2021). A further review reported that 70% of studies conducted with sexual and gender minorities found reductions in sexual behavior (Kumar et al., 2021). A survey of 1559 adults in the USA showed that the social distancing measures led to the decline of sexual behaviors in 44% of the sample, and an improvement in sex life for 14% (Lehmiller et al., 2021). The general decline in sexual activities, not associated with age, gender or socioeconomic status, was also reported in regard to individual sexual practices such as masturbation. There was, however, an increase in non-physical sexual activities such as sexting, having cybersex, sending nude photographs, or watching pornography, which was significantly higher in sexual minorities.

A similar decline in sexual behaviors, desire and arousal was reported in other countries affected by lockdown measures: a survey from Italy showed mixed effects on sexual desire with 18% of men and 26% of women having less desire, whilst 12% of men and 19% of women reported an increase (Panzeri et al., 2020). In a Spanish study, a third of participants (36%) reported an increase in sexual desire and similarly a third (35%) reported a decline (Ballester-Arnal et al., 2021). A UK-based survey of 868 respondents reported that during lockdown 40% of the sample engaged in any sexual activity at least once per week on average (Jacob et al., 2020). Those sexually active were more likely to be men, of younger age, employed, in a domestic relationship, and of higher annual income, with an average number of sexual activities of 1.75. However, the survey did not measure sexual orientation and the number of non-steady partners, which could provide additional insights into the impact of social distancing measures on sexual behaviors on specific communities.

Men (cis and trans) and gender diverse people who have sex with men (MGDSM) are at higher risk of sexually transmitted infections (STIs). For cis-men who have sex with men (MSM), this is well documented in expansive literature. In the UK, STI diagnoses have increased by 5% between 2018 and 2019 (447,522 to 468,342) with gonorrhea and syphilis showing the highest rates since records began in 1918 (Mitchell et al., 2020). Between 2015 and 2019, MSM demonstrated large increases in diagnoses of chlamydia (83%; from 12,687 to 23,187), gonorrhea (51%; from 22,413 to 33,853), and syphilis (40%; from 4,183 to 5,875) and continue to be disproportionately affected by STIs. Gender diverse people who have sex with men are poorly represented in HIV prevention and sexual health research generally. However, evidence suggests trans men, trans women and non-binary people are less likely to frequently test for HIV and STIs and face pronounced barriers to service access both in the UK and globally, making them a priority group in the HIV response (Hibbert et al., 2020; Scheim et al., 2016; Winter et al., 2016); Witzel et al., 2021). Therefore, changes in sexual behaviors in this group have a significant impact on sexual health services across the UK and any change in sexual risk-taking is needed to help plan strategies to help ameliorate any increases in infections.

### Current Study

Both sexual activity (including casual sex) and good sexual health are an important component of well-being for many, and it is unlikely that government restrictions would universally halt sex with new partners. Casual sex is also of specific interests during social distancing mandates as it is more likely to involve individuals outside of one’s own household, with clear links for transmission of SARS-CoV-2. In addition, casual sex is often associated with greater potential for transmission of HIV and other STIs (Vivancos et al., 2010). Coupled with closure of some sexual health services and severe limitations placed on other services during the first UK lockdown, it is imperative to understand which groups were most likely to maintain or increase sexual activity with casual partners in order to effectively target sexual health services to those with the greatest need. This study therefore aimed to explore the self-reported changes in sexual behaviors in MGDSM in the UK to inform the development and delivery of sexual health services and to identify those in need of tailored risk reduction interventions during and following the COVID-19 pandemic.

## Method

This online cross-sectional survey assessed engagement in casual sex (i.e., sex with unestablished non-steady sexual partners) during the first UK-wide lockdown of the COVID-19 pandemic among MGDSM (23 March to 15 June 2020). Correlates of engagement in casual sex were also explored. A purposefully broad definition of casual sex was used in order to reflect the range of potential understandings within this diverse population. The study received the approval of the University of University of Westminster Research Governance and Ethics Committee (reference: ETH1920-1601) and from the London School of Hygiene and Tropical Medicine Observational Research Ethics Committee (reference: 22421).

### Participants

We aimed to explore the sexual behavior of gender diverse people (i.e. trans and cis-men as well as trans women and non-binary people) who have sex with men. These included men who identify as gay, bisexual, or queer. All participants needed to be 18 years or older and living in the UK. Those who reported having sex exclusively with women were excluded from the analysis.

Between 20 April and 22 May 2020, an online study advert was distributed through social media (Twitter, Facebook and Instagram) inviting MGDSM to take part in a survey exploring the use of hook-up apps during the lockdown. Recruitment used convenience and snowball sampling approaches. A combination of various MSM and gender minority-related hashtags and handles were applied to boost online recruitment. In addition, the hook-up app Grindr provided a pro-bono promotion to their UK users, in the form of a pop-up message, to reach MSM and other gender diverse groups. As no physical contact was permitted during this time, recruitment focused on individuals on various digital channels and the views of those who were not on the Internet were not gathered. All participants were invited to enter a prize draw worth £75. When clicking on the advert, participants were directed to the online survey that contained the participant information page outlining the aims of the study and a consent page.

### Measures and Procedure

The survey consisted of 40 demographic, behavioral and attitudinal questions (Supplementary file A) hosted on Qualtrics. Upon completion, participants were presented with a debriefing web page outlining information about sexual health and COVID-19 with links to online resources and UK providers of sexual health services.

The survey included eight demographic questions: age, country of residence (England, Northern Ireland, Scotland, Wales), gender identity (male [including trans male], female [including trans female], non-binary), sex assigned at birth (male, female, indeterminate), the gender of sexual partners (male [including trans male], female [including trans female] non-binary), education level (standard UK education codes), ethnicity (standard UK ethnicity codes), and relationship status (single/in a monogamous relationship/in an open relationship/it’s complicated/other). Three behavioral questions assessed whether participants ever exchanged sex for money or goods (yes/no); if they had been able to be with their main sexual partners during the lockdown (yes/no); and with whom they shared their household during the lockdown (no one/parents or family members/romantic partner/regular house or flat mates/children/other. Ten healthcare-related questions asked about HIV status; HIV PrEP use before lockdown; HIV PrEP intermission during the lockdown; reasons for interrupting HIV PrEP as well as access to STI screening; STI test results and STI treatment since the beginning of lockdown. Two questions assessed perceived COVID-19 infection and testing uptake. Further risk indicator questions explored the frequency and usage of sexual networking phone applications (e.g., Grindr, Scruff, Tinder, or any other). Six questions, with options “reduced a lot,” “reduced a bit,” “stayed the same,” “increased a bit,” and “increased a lot,” examined the change in sexual behaviors such as the usage of sexual networking phone applications; the engagement in virtual sex such as webcam sex, phone sex, sexting or exchange of naked pictures; looking online for a steady, ongoing partner or a boyfriend; the time spent chatting to people on sexual networking phone applications; the engagement in casual sex with one person; and the engagement in casual sex with three or more people (e.g., threesome or group sex). Participants were asked to type the number of casual sex partners they met for sex since the beginning of the UK lockdown on the 24 of March 2020. Next, the participants were asked how long they were able to abstain from casual sex and if they re-engaged with sexual partners during the lockdown, reporting the reasons for meeting sexual partners during the lockdown. One question asked about methods used by those participants to reduce the transmission of the SARS-CoV-2 virus. MGDSM were also asked how much time they had been feeling anxious about the ongoing outbreak and about contracting the virus through casual sex, with six responses ranging from “at no time” to “all of the time.” Finally, a short version of the Warwick–Edinburgh Mental Well-being Scale (Tennant et al., 2007) was completed to assess emotional health and distress.

### Data Analysis

Our exploratory approach to data analysis aimed to identify variables that had a statistically significant relationship with our outcome variable; thus, no prior hypotheses were tested (Sperandel, 2014). Our outcome variable was the number of casual sex partners during the lockdown, dichotomized into “zero/no casual sex” and “one or more casual sex partners.” Following consultation with those working in clinical and voluntary sectors, we used a binary variable to enhance our ability to identify statistically significant identify differences across sub-groups. This enables improved targeting of sexual health services to the broad groups most likely to have need, acknowledging that a more nuanced approach would produce more granular findings (but at the expense of statistical significance).

All raw data were transferred from Qualtrics into IBM SPSS software version 24 for analysis. The dataset was processed and cases that did not meet inclusion/exclusion criteria were removed. After applying inclusion and exclusion criteria variable distributions were checked for outliers, extreme values and invalid responses. The proportion of missing data varied by the type of measurement, due to questionnaire routing, but did not exceed 20% within the dataset (11% for the outcome variable). Missing variables were handled by the SPSS software and all cases analyses were performed. Descriptive statistics were applied to identify the distribution of responses. Logistic regressions were performed with a single categorical predictor, and statistically significant predictors, at *p* < 0.05, were entered into a multivariable model for adjustment.

## Results

### Sample Characteristics

In total, 1429 participants completed the survey, with a mean age of 36 (Table 1). The majority identified as ‘male or trans male’ (97%) and were assigned male sex at birth (98%), with 29 participants (2%) identifying as female or non-binary. While most had sex with only men, 7% reported having sex with both men and women. Around 85% identified as white and 65% indicated having a higher education degree. Approximately 68% were single, 31% were the lone member of their household during the lockdown and 2% reported exchanging sex for money or goods. Fifty-six percent did not think they had a previous SARS-CoV-2 infection, 2% were tested for the virus. Within the sample, 12% were HIV positive and 29% reported using PrEP before the lockdown. Among PrEP users, 69% reported discontinuing PrEP during the lockdown. About 12% accessed STI screening during the lockdown, mostly using self-testing or self-sample kits (73%). Most participants reported not feeling anxious about the ongoing outbreak (61%) or contracting the SARS-CoV-2 virus through casual sex (69%).

**Table 1:** Sample characteristics

Variable	N	(%) or [mean, SD]	Variable	N	(%) [mean, SD]
**Demographic variables—Total N**	1429		**Sexual behaviors during to lockdown**		
Age		[36.6, 11.4]	Frequency of sexual networking app use		
Gender identity			Every day	871	(66)
Male (including trans male)	1386	(97)	Several times per week	255	(19)
Female (including trans female)	6	(< 1)	Once per week or less often	189	(15)
Non-binary	28	(2)	Change in sexual networking app use		
Other	9	(< 1)	Reduction	556	(42)
Gender at birth			Stayed the same	288	(22)
Male	1407	(98)	Increase	464	(36)
Female	22	(2)	Change in ‘virtual sex’ (e.g. sexting)		
Sexuality (gender of sexual partners)			Reduction	317	(24)
Men (including trans men)	1317	(92)	Stayed the same	544	(42)
Both men and women	102	(7)	Increase	450	(34)
Prefer not to say	10	(1)	Change in seeking for a steady partner		
Ethnicity			Reduction	396	(30)
White	1207	(85)	Stayed the same	629	(48)
Mixed/multiple ethnic groups	48	(3)	Increase	278	(22)
Asian/Asian British	69	(5)	Change in casual sex with one person		
Black/African/Caribbean	47	(3)	Reduction	1014	(78)
Other: Hispanic/Latino	39	(3)	Stayed the same	249	(19)
Other: Arab/any other	18	(1)	Increase	44	(3)
Education			Change in casual sex with 3 or more people		
No secondary qualification	6	(< 1)	Reduction	794	(62)
GCSE or equivalent	115	(8)	Stayed the same	468	(36)
A-level or equivalent	196	(14)	Increase	31	(2)
Higher education below degree	158	(11)	Number of casual sex partners in lockdown		
Degree or higher	923	(65)	Zero	972	(76)
Other	30	(2)	One	155	(12)
Relationship status			Two	53	(4)
Single	965	(68)	Three	37	(3)
In a monogamous relationship	113	(8)	Four or more	65	(5)
In an open relationship	223	(16)	Ability to refrain from sexual contact		
It’s complicated	108	(7)	No time at all/no ability	11	(1)
Other	18	(1)	Up to two weeks	12	(1)
Perceived past infection with SARS-CoV-2			Up to four weeks	94	(10)
Yes	185	(14)	Up to three months	261	(27)
No	730	(56)	Up to six months or longer	549	(61)
Not sure	400	(30)	Reasons for casual sex during the lockdown*		
Screened for SARS-CoV-2			Feeling horny	212	(68)
Yes	22	(2)	Feeling lonely/wanting intimate contact	127	(40)
No	1292	(98)	Bored/needed distraction	57	(18)
Experience of lockdown			Feeling stressed/sex helps relax	45	(14)
Alone	446	(31)	Needing to get away from living place	33	(10)
With a parent/family	317	(22)	Being in a cruising ground	17	(5)
With a partner	289	(20)	Being pressured to meeting sex partners	5	(1)
With a housemate	360	(25)	Needed to make money through sex work	4	(1)
With a child	25	(2)			
Ever exchanged sex for money (sex work)			**Emotional health variables**		
Yes	26	(2)	Time feeling anxious about the outbreak		
No	1391	(97)	At no time	150	(12)
Prefer not to say	12	(1)	Some of the time	614	(49)
HIV status			Less than half of the time	154	(12)
Diagnosed	169	(12)	More than half of the time	266	(21)
Never diagnosed	1250	(87)	All of the time	65	(6)
Not sure/prefer not to say	8	(< 1)	Time feeling anxious about getting SARS-CoV-2 via sex		
PrEP use before the lockdown (January 2020)			At no time		
Yes	362	(29)	Some of the time	513	(41)
No	883	(71)	Less than half of the time	350	(28)
PrEP intermission during to lockdown			More than half of the time	59	(5)
Yes	247	(69)	All of the time	191	(15)
No	111	(31)	Warwick–Edinburgh Mental Well-being Scale	131	(11)
STI screening during the lockdown			Average or high wellbeing		[22.4, 4.96]
Accessed	173	(12)	Possible or probable depression	605	(50)
No accessed	1242	(88)		601	(50)
STI screening method					
Physically in a clinic	39	(23)			
Using a self-test/sample kit	126	(73)			
At a GP practice	1	(< 1)			
Other	6	(3)			

*Multiple answers within the subgroup that engaged in casual sex; STI—sexually transmitted infection; PrEP—pre-exposure prophylaxis

### Sexual Behaviors During the Lockdown

Eighty-five per cent (*n* = 1126) of participants reported using hook-up apps every day or several times per week during the first UK-wide lockdown, with 36% reporting an increase in app usage. Approximately, 76% self-reported similar or increased use of virtual sex, such as webcam sex or sexting, and 70% used apps to look for a steady partner or a boyfriend. While 78% reported a reduction in casual sexual intercourse, 3% indicated an increase in comparison to the time before the lockdown. Similarly, 62% reported a reduction in seeking sex with multiple partners and 2% indicated an increase. Three-quarters of the sample (76%) self-reported not engaging in casual sex during the lockdown, with 12% reporting meeting one casual sex partner and 5% engaging in casual sex with multiple partners. Twenty-four per cent of men (cis and trans), 28% of non-binary people and none of those identifying as female engaged in casual sex. The main reasons for engaging in casual sex were: feeling horny (68%); feeling lonely and wanting intimate contact (40%); and being bored or needing distraction (18%). Twelve per cent thought they were able to refrain from sexual contact for up to four weeks during the lockdown, and 61% were able to refrain for up to six months.

The analysis revealed significant correlates of casual sex during the lockdown (Table 2). The strongest predictors of engaging in casual sex in univariate regressions were the exchange of sex for money and goods, and reported increases in casual sex with one and multiple sex partners. However, these variables were excluded in the adjusted models due to the small count. Living circumstances during lockdown was associated with sexual activity with 29% of those living alone reporting having casual sex, compared to those living with parents (15%), a romantic partner (19%) or children (22%), but the variable was no longer significant after adjustment. Lower education, either having GSCE or no formal qualification (40%), was associated with engaging in casual sex, compared to those having a university degree (23%). Identifying as an ethnic minority was also a significantly correlate, with 37% of Black or Black British, 32% of Asian or British Asian, 36% of mixed-race, 39% of Hispanic/Latinx, and 33% of other ethnicities reporting engaging in casual sex, compared to 22% for those identifying as white. PrEP use was a significant predictor of casual sex during the lockdown, with 45% of those who used PrEP reporting that they engaged in casual sex. Also, a significantly larger proportion of participants (30%) who used PrEP before lockdown but intermitted it engaged in casual sex. Those who used sexual networking apps daily and those who were less anxious about being infected with SARS-CoV-2 via sex were more likely to engage in casual sex.

**Table 2:** Factors associated with the engagement in casual sex during the UK lockdown (March–April 2020) in men and gender diverse people who have sex with men

Variable	*N* (%)	*N* (%) engaged in casual sex	Crude OR [95% CI]	Adjusted OR [95% CI] ¥
Age				
< 25	212 (17)	45 (21.2)	1.01 [0.65–1.55]	
26–35	435 (34)	117 (26.9)	1.38 [0.97–1.95]	
36–45	332 (26)	85 (25.6)	1.29 [0.89–1.86]	
46 +	304 (23)	64 (21.1)	Ref	
Gender identity				
Male (including trans men)	1386 (97)	301 (24.2)	0.89 [0.42–1.85]	
Other (non-binary)	43 (3)	10 (26.3)	Ref	
Education				
GSCEs or no qualification	107 (8)	43 (40.2)	2.26 [1.49–3.44]*	2.37 [1.40–4.01]*
A level or equivalent	171 (14)	32 (18.7)	0.77 [0.51–1.17]	0.95 [0.57–1.57]
Higher education but not degree	146 (12)	39 (26.7)	1.20 [0.82–1.83]	1.20 [0.78–1.09]
Degree or equivalent	831 (66)	190 (22.9)	Ref	Ref
Ethnicity				
Ethnic minority	182 (13)	54 (34.6)	1.84 [1.28–2.64]*	2.27 [1.40–3.53]*
White	1207 (87)	243 (22.3)	Ref	
Sexuality				
Sex with both men and women	102 (8)	25 (27.8)	1.20 [0.74–1.95]	
Sex exclusively with men	1317 (92)	286 (24.1)	Ref	
Relationship status				
Single	873 (69)	215 (24.6)	1.26 [0.72–2.20]	
It’s complicated	103 (8)	26 (25.2)	1.31 [0.65–2.62]	
In an open relationship	210 (17)	50 (23.8)	1.21 [0.65–2.25]	
In a monogamous relationship	83 (6)	17 (20.5)	Ref	
Experience of lockdown				
Alone	444 (31)	120 (29.1)	1.46 [1.12–1.91]*	1.21 [0.87–1.69]
Sharing household	965 (69)	191 (21.9)	Ref	Ref
Ever exchanged sex for money				
Yes	446 (31)	16 (72.7)	8.75 [3.39–22.58]*	
No	983 (69)	292 (23.3)	Ref	
HIV status				
Diagnosed	26 (2)	38 (25.3)	1.08 [0.73–1.60]	
Never diagnosed	1391 (98)	268 (23.8)	Ref	
PrEP use				
Used before lockdown, but intermitted	233 (21)	69 (29.6)	1.76 [1.26–2.46]*	1.75 [1.20–2.56]*
Continuous PrEP use during lockdown	103 (9)	46 (44.7)	3.38 [2.20–5.18]*	2.79 [1.76–4.57]*
No prior PrEP use	784 (70)	151 (19.3)	Ref	Ref
STI screening during lockdown				
Yes	161 (13)	70 (43.5)	2.82 [2.00–3.98]*	2.65 [1.76–3.99]*
No	1117 (87)	239 (21.4)	Ref	Ref
Perceived past infection with SARS-CoV-2				
Yes	184 (14)	53 (28.8)	1.41 [0.98–2.03]	
Not sure	393 (31)	101 (25.7)	1.21 [0.90–1.61]	
No	706 (55)	157 (22.2)	Ref	
Frequency of sexual networking app use				
Every day	185 (13)	237 (27.8)	1.85 [1.38–2.48]*	2.24 [1.54–3.25]*
Several times per week or less often	1130 (86)	74 (17.2)	Ref	Ref
Change in sexual networking app use				
Increased	451 (36)	126 (27.9)	1.59 [1.18–2.14]*	
Stayed the same	284 (22)	73 (25.7)	1.42 [1.01–2.00]*	
Reduced	542 (42)	106 (19.6)	Ref	
Change in ‘virtual sex’ (e.g. sexting)				
Increased	440 (34)	115 (26.1)	1.07 [0.76–1.50]	
Stayed the same	534 (42)	120 (22.5)	0.88 [0.63–1.22]	
Reduced	307 (24)	76 (24.8)	Ref	
Change in seeking a steady partner				
Increased	272 (21)	75 (27.6)	1.43 [0.99–2.05]	
Stayed the same	615 (48)	153 (24.9)	1.24 [0.91–1.69]	
Reduced	386 (31)	81 (21.0)	Ref	
Change in casual sex with 3 or more people				
Increased	30 (2)	27 (90.0)	29.7 [8.91–99.1]*	
Stayed the same	454 (36)	97 (21.4)	0.89 [0.67–1.18]	
Reduced	779 (62)	181 (23.2)	Ref	
Change in casual sex with one person				
Increased	43 (3)	41 (95.3)	74.3 [17.8–309.9]*	
Stayed the same	239 (19)	54 (22.6)	1.05 [0.75–1.48]	
Reduced	995 (78)	215 (21.6)	Ref	
Anxious about getting SARS-CoV-2 via sex				
Half of the time or less	922 (74)	230 (24.1)	1.56 [1.13–2.16]*	1.66 [1.12–2.44]*
More than half of the time	322 (26)	56 (17.7)	Ref	Ref
Warwick–Edinburgh Mental Well-being Scale				
Lower wellbeing/probably depression	592 (49)	139 (23.5)	1.08 [0.82–1.41]	
Average or high wellbeing	602 (51)	133 (22.1)	Ref	

^*^Significant at p < 0.05; STI—sexually transmitted infection; PrEP—Pre-exposure prophylaxis against HIV; ¥ variables included in the adjusted model (education, ethnicity, experience of lockdown, PrEP use, STI screening during lockdown, frequency of app use, anxious about getting SARS-COV-2 via sex); variables excluded from the adjusted model due to small count (exchange money for sex, change in casual sex with one person, change in casual sex with 3 or more people)

## Discussion

This is the first study examining the impact of UK-wide lockdown on sexual behaviors in MGDSM. Despite high activity on sexual networking apps and an increase in “virtual” non-physical sex, three-quarters of the sample reported suspending any sexual activities with non-steady partners, indicating a major shift in sexual behaviors in response to the COVID-19 social distancing measures. Despite public health messages against household mixing during the first UK lockdown, a quarter of MGDSM engaged in casual sex, with a small proportion of 3% reporting a substantial increase in sex-seeking behaviors, demonstrating a need for accessible sexual health services offering STI testing and treatment. Although motivations for casual sex varied, many participants reported feeling horny, lonely and lacking social contact, suggesting the potential role of psychosocial stress in engagement with casual sex and highlighting the need for a nuanced and non-judgmental view on sexual activity during this period, despite its illegality at the time. This is consistent with research from Brazil and Portugal which reported MSM engaged in casual sex partly because of social isolation (de Sousa et al., 2021).

Although the number of participants reporting exchanging money for sex was small (*n* = 26) and the confidence interval for this association was wide, receiving money for sex was identified as the strongest predictor, demonstrating the association between economic need and health vulnerability. This highlights the tension of public health calls for widespread sexual abstinence and physical distancing during the lockdown. A lack of nuance between those who were able to adhere to guidelines, and those who ceased contact with others presents considerable challenges concerning economic survival (Platt et al., 2020). Although the pandemic had a negative impact on global male sex work, tailored education and outreach are needed to support individual and public health (Callander et al., 2020). Parallel narratives might be drawn between those undertaking other work (i.e. not sex work) who, despite calls to stay at home, had few options other than to continue working, thereby increasing their COVID-19 risk. Future public health strategies relating to COVID-19 or other infectious disease outbreaks need to consider the specific needs of those undertaking sex work. These should include the needs of those who are unable to access the social support or healthcare system because of fear or actuality of incarceration or deportation.

Consistent with research from the USA, respondents from ethnic minority groups were more likely to engage in casual sex than white participants, highlighting the need for culturally relevant health promotion to reach these groups with practical risk reduction advice (Schumacher et al., 2022). Systematic review evidence suggests that MSM from minority ethnic and migrant backgrounds have increased needs surrounding HIV prevention and sexual health. These are potentially compounded by syndemic associations between HIV risk factors and drug or alcohol use (Lewis & Wilson, 2017). Experiences of homophobia and racism, discrimination on account of sexual orientation and financial hardship in ethnic minority MSM have previously been associated with psychological distress and participation in unsafe sexual situations (Díaz et al., 2004). As such, future research should identify the comprehension of health promotion regarding sex during the COVID-19 pandemic in ethnic minority MGDSM and whether distress, financial stability or the experiences of discrimination can explain engagement in casual sex.

Lower education was also associated with engagement in casual sex. This finding is in line with wider literature demonstrating the association between the risk of STIs and HIV and education, especially for those from the most disadvantaged populations (Janssen et al., 2000). MGDSM with lower educational qualifications experience socioeconomic disconnection, with limited access to education and employment opportunities and associated lower literacy around health risks, STIs and SARS-COV-2 (Gayles et al., 2016). Public health policies need to acknowledge challenges experienced by the most disadvantaged social groups when designing interventions to minimize risks of infections.

There was a substantial interruption to PrEP use during the first UK lockdown. Encouragingly, participants who utilized sexual health services such as STI testing and continued taking PrEP were more likely to engage in casual sex. It is, however, concerning that a significant number of participants who discontinued PrEP during lockdown engaged in casual sex. Further, previous research highlights that the pandemic-related restrictions were associated with lower utilization of PrEP during lockdowns (Reyniers et al., 2021) and that many who attempted to access PrEP in the Republic of Ireland during the COVID-19 crisis were not able to. However, it is uncertain how many MGDSM in our sample experienced barriers to accessing PrEP use as well as potential interruptions to services required to restart PrEP safely (see Brady et al., 2019), while social distancing measures were in place.

The majority of participants showed low levels of anxiety about the ongoing SARS-CoV-2 pandemic or contracting the novel coronavirus via sex, with 1 in 10 perceiving themselves to have already been infected. Indeed, anxiety was a key predictor of abstention from casual sex during this period. It is likely that as the COVID-19 crisis normalized over time, people will have become less anxious about the infection and the engagement in casual sex will have thus increased. It is important to provide useful and appropriate risk reduction information to enable informed sexual decision making for MGDSM. Subsequent qualitative studies should explore the perceptions of STI risk during the pandemic to identify any factors associated with self-protective behaviors.

Although comparable data for gender diverse people are scant, similar findings were identified in other studies amongst MSM. A survey of 694 MSM in Belgium showed a significant decline in physical sex with non-steady partners from 59 to 9% during the first weeks of lockdown (Reyniers et al., 2021). Casual sex was associated with HIV-positive status and PrEP use, as well as engagement in group sex, chemsex and sex work. In that sample, 47% stopped using PrEP and many had concerns about follow-up PrEP care appointments during the pandemic. A survey of 1051 MSM with a median age of 35, collected in the USA in April 2020, showed a 68% decline in opportunities to have sex and a 51% decrease in the number of sex partners (Sanchez et al., 2020). The study also reported an increase in the use of hook-up apps to connect with men but not to meet in person, with only 4% reporting an increase in casual sex. One online survey of 518 MSM in the USA showed that 67% believed it was possible to contract COVID-19 through sex and kissing, with a moderate willingness to engage in sex (Stephenson et al., 2021). Interestingly, the study reported an increase of 2.3 sex partners during the lockdown, associated with older age (25–44), a higher level of education, ‘other’ sexual identity, HIV-negative status, and drug use, in contrast with our findings that MGDSM with lower educational attainment were more likely to engage in casual sex. Another survey of 1001 MSM in the USA reported a decline in condomless anal sex from 72 to 26% due to COVID-19; however, the number of casual sex partners, with an average of 1.5, did not differ significantly, indicating a change in the type of sexual activities during lockdown (Starks et al., 2020).

There are also reports indicating a decline in sexual health service utilization, such as an 80% drop in PEP prescription in a London clinic (Juneho et al., 2020), a 78% decline in the Madrid region of Spain (Sanchez-Rubio et al., 2021), and 66% in Melbourne for the duration of the lockdown (Chow et al., 2020). Also, an assessment of PrEP users’ sexual behaviors in Wales showed a significant decline in condomless sex from 42 to 19% due to lockdown, with the sharpest decline to 13% in men who declared being single (Gillespie et al., 2021). Therefore, although many MDGSM refrained from engaging in casual sex while observing strict social distancing measures, a substantial proportion remained sexually active for a variety of reasons. Further, a recent study of young adults in Australia found declining condom use during the COVID-19 pandemic (Dacosta et al., 2021). While we did not measure this in our sample, should this also be true in the UK, this will enhance the need to res-establish and expand testing for HIV and other STIs. Taken together, our results and data on health service utilization indicate the need to re-establish and expand sexual health services offering testing for HIV and other STIs.

### Methodological Limitations and Future Considerations

There are several methodological considerations. Unlike previous studies, our survey included gender diverse people providing insight into behaviors of sexual and gender minorities. The data collection began one month after the lockdown was imposed, for one month only, thus reflecting the views of participants at that time. There is a possibility that the longer duration of data collection could identify more MGDSM engaging in casual sex due to the normalization of the restrictions and fatigue. Future research could measure this over a greater period of time, and measure changes based on survey completion date.

We also did not measure whether our participants adhered to lockdown restrictions generally and to what extent the observed self-reported changes in sexual behaviors are due to lockdown and the ongoing pandemic. This means that some of the observed changes could be related to other factors (e.g. anxiety related to the pandemic rather than government restrictions themselves or from natural lulls in sexual activity); we also cannot assess whether individuals who had casual sex generally ignored government restrictions in their entirety. Future research assessing similar phenomena could include these elements and produce a more nuanced analysis by examining motivational factors.

In addition, our cross-sectional survey did not measure the engagement in casual sex before the pandemic for a precise estimation of change and there is a chance that some participants had not been sexually active, with small increases in sexual activity thus potentially confounding the analysis. As the study is cross-sectional, the relationship between variables is correlational and causality could not be determined from this analysis. Future research could ask about prior sexual activity and make a comparison, or take a cohort approach, facilitating more robust measures of change and enhancing the analysis of potential need.

The sample was recruited through social media and a popular hook-up app and may overestimate the proportion of MGDSM engaging in casual sex, as the views of those who were not users of sites for sexual networking were not explored. This may result in self-section bias. However, our recruitment strategy could be justified due to social distancing measures being in place during data collection. Conversely, participants were asked about sexual activities, which were deemed prohibited for the duration of the lockdown, hence some participants may have hesitated to reveal the number of casual sex partners. Thus, there is a possibility of social desirability bias. The survey did not explore the impact of lockdown on sexual behaviors for those in steady relationships, instead, the analysis focused on casual sex only, offering a limited understanding of the change in sexual behaviors. Our survey lacked a measure of geographical location to determine whether our sample was drawn from rural or urban areas. There is a possibility that our recruitment strategy attracted responses from MGDSM from large cities with greater access to sexual networks and opportunities for casual sex.

Future research should also assess whether MGDSM experienced greater barriers to accessing sexual health services during the pandemic, with a focus on sexually active individuals and to identify the drivers of continued sexual behavior during times of social distancing measures. Qualitative studies may explore the impact of social distancing measures on the experiences of intimacy and connection, providing insight into the impact of lockdown on mental health, chemsex participation and dating. Our study emphasizes the need to provide clear messages about the accessibility of sexual health services, risk reduction strategies against STIs and SARS-CoV-2, as well as offer support services for MGDSM experiencing loneliness and anxiety.

### Conclusion

In our study of 1429 MGDSM, we found that the majority of individuals reported reductions in sexual activity during the first UK COVID-19 lockdown. In self-reported data, 78% reported reductions in casual sexual intercourse, with a small proportion (5%) reporting increases. Those who reported having engaged in casual sex in this period were more likely to have low educational attainment, identify as an ethnic minority, be taking PrEP, use hook-up apps daily and be less anxious about SARS-CoV-2 transmission. A substantial proportion of those taking PrEP who halted it during lockdown (30%) engaged in casual sex. Our results indicate potential unmet need, with those already experiencing sexual health inequalities in the UK most likely to have ongoing utility for health promotion and clinical intervention.

## Supplementary Information

Below is the link to the electronic supplementary material.

Supplementary file1 (DOCX 29 kb)

## Data Availability

Data are available upon request.

## Declarations

### Editor’s note

This paper was to be part of the Special Section on the Impact of COVID-19 on Sexual Health and Behavior (Guest-Edited by Lori A. J. Scott-Sheldon, Kristen P. Mark, Rhonda N. Balzarini, and Lisa L. M. Welling), which was published in the January 2022 issue of the Journal. Unfortunately, the paper was not accepted for publication until after the January 2022 issue went to press.
