# Supplementary material for: The Impact of First UK-Wide Lockdown (March–June 2020) on Sexual Behaviors in Men and Gender Diverse People Who Have Sex with Men During the COVID-19 Pandemic: A Cross-Sectional Survey
Source: Arch Sex Behav. 2022 Nov 7;52(2):617–27. doi: 10.1007/s10508-022-02458-6 (PMC9640839; doi:10.1007/s10508-022-02458-6)
Supplement: Supplementary file 1 — Supplementary file1 (DOCX 29 kb) [file 10508_2022_2458_MOESM1_ESM.docx]

**The impact of first UK-wide lockdown (March-June 2020) on sexual behaviours in men and gender diverse people who have sex with men during the COVID-19 pandemic: A cross-sectional survey**

Supplementary file A

Do you currently live in the UK?

- Yes (1)
- No, please specify (2) ________________________________________________

Have you used at least one sexual networking or dating app (e.g. Grindr, Scruff, Tinder, or any other) in the last year?

- Yes (1)
- No (2)

What's your age (in years)?

________________________________________________________________

How do you identify?

- Male (including trans male) (1)
- Female (including trans female) (2)
- Non-binary (3)
- Other (4)

What sex were you assigned at birth?

- Male (1)
- Female (2)
- Undetermined (3)

What is your highest educational qualification?

- Degree, equivalent, or higher (1)
- Higher education (2)
- A-level, or equivalent (3)
- GCSEs grades A* to C, or equivalent (4)
- Other qualifications (5)
- No qualifications (6)

What is your ethnic background?

- White: English/Welsh/Scottish/Northern Irish/Traveller/Any other White background (1)
- Black: Black British/African/Caribbean/Any other Black background (2)
- Asian: Asian British/Indian/Pakistani/Bangladeshi/Chinese/Any other Asian background (3)
- Mixed: White and Black Caribbean/White and Black African/Any other Mixed background (5)
- Other: Arab/Any other ethnic group (6)
- Other: Hispanic/Latino (7)

Who do you usually have sex with?

- Men (incl. trans men) (1)
- Women (incl. trans women) (2)
- Both men and women (3)
- Prefer not to say (4)

How many people are in lock-down with you at your address?

- No one/ I am alone at the moment (1)
- Parents and/or other family members (2)
- Romantic partner(s) (3)
- Your regular house/flat-mates (4)
- Children (5)
- Other (6)

What is your relationship status?

- Single (1)
- In a monogamous relationship (2)
- In an open relationship (3)
- It’s complicated (4)
- Other (5)

Are you currently able to be with your main sexual partner since social distancing measures due to the coronavirus began in March 2020?

- Yes (1)
- No (2)
- Not applicable (3)

Have you exchanged sex for money or goods since social distancing measures due to the coronavirus began in March 2020?

- Yes (1)
- No (2)
- Prefer not to say (3)

Have you ever been diagnosed with HIV by a health professional?

- Yes (1)
- No (2)
- Prefer not to say (3)
- Not sure (4)

Have you taken PrEP at any time through January 2020 (before the coronavirus outbreak)?

- Yes (1)
- No (2)
- Prefer not to say (3)

Have you interrupted your regular PrEP since January 2020?

- Yes (1)
- No (2)
- Prefer not to say (3)

Why did you interrupt your PrEP? (please tick all that apply)

- Side effects of PrEP medicine (1)
- I was in a monogamous (or closed polyamorous) relationship (with no perceived HIV risk) (2)
- I was/I am not having sex at the moment (3)
- I was/I am using condoms all the time (4)
- I was/I am having sex but didn't feel at risk (5)
- I forgot to take my PrEP pills (6)
- Due to health issues/Impact on my sex life (7)
- I wanted a break from PrEP (8)
- I experienced PrEP-related stigma (9)
- I didn't have any PrEP left and I can no longer access PrEP supply from the IMPACT or other trials (10)
- I didn't have any PrEP left and I can no longer access PrEP from my health service provider (11)
- I didn't have any PrEP left and I can no longer access PrEP from an online supplier (12)
- I didn't have any PrEP left and I can no longer afford to buy PrEP (13)
- I didn't have any PrEP left and restrictions on travel in the UK make it hard for me to get PrEP (14)

Have you accessed any STI testing since social distancing measures began in March?

- Yes (1)
- No (2)
- Not sure (3)

How/where did you get this STI test?

- Physically in a clinic (1)
- Through using a self-test kit (2)
- At a GP practice (3)
- Other, please specify (4) ________________________________________________

Have you accessed STI test results since social distancing measures began in March?

- Yes (1)
- No (2)
- Not sure (3)

How/where did you get these STI results?

- Physically in a clinic (1)
- Through using a self-test kit (2)
- Via a telephone or video-call with a healthcare professional (3)
- Via an online service (6)
- Other, please specify (7) ________________________________________________

Have you received medication to treat an STI since social distancing measures began in March?

- Yes (1)
- No (2)
- Not sure (3)

How/where did you get this STI medication?

- Physically in a clinic (1)
- Collected prescription from a local pharmacy (2)
- Received medication by post (6)
- Other, please specify (7) ________________________________________________

Do you think you have been infected with the novel coronavirus at any point in the last few months?

- Yes (1)
- No (2)
- Not sure (3)
- Prefer not to say (4)

Have you had a test for the novel coronavirus?

- Yes (1)
- No (2)
- Not sure (3)

**Now, we are interested in the use of sexual networking apps** (e.g. *Grindr*, *Scruff*, *Tinder*, or any other) **since social distancing measures due to the novel coronavirus began in March 2020.**    **Please answer the next few questions with the option that best represents your experience:**

How often have you opened a sexual networking app (e.g. *Grindr*, *Scruff*, *Tinder*, or any other) in the time since social distancing measures due to the coronavirus began in March 2020?

- Several times a day (1)
- Every day (2)
- Several times per week (3)
- Once a week (4)
- Several times per month (5)
- Once per month (6)
- Less than once a month (7)

Has the number of occasions you opened a sexual networking app (e.g. *Grindr*, *Scruff*, *Tinder,* or any other) changed since social distancing measures began in March 2020?

- Reduced a lot (1)
- Reduced a bit (2)
- Stayed the same (3)
- Increased a bit (4)
- Increased a lot (5)

Has the number of occasions you had sex without physical contact  (e.g. webcam sex, phone sex, sexting, exchange of naked pictures) changed since social distancing measures began in March 2020?

- Reduced a lot (1)
- Reduced a bit (2)
- Stayed the same (3)
- Increased a bit (4)
- Increased a lot (5)

Has the amount of time you spend looking online for a steady, on-going partner or boyfriend changed since social distancing measures began in March 2020?

- Reduced a lot (1)
- Reduced a bit (2)
- Stayed the same (3)
- Increased a bit (4)
- Increased a lot (5)

Has the amount of time you spend just chatting to people on apps (without intending to arrange a hook-up) changed since social distancing measures began in March 2020?

- Reduced a lot (1)
- Reduced a bit (2)
- Stayed the same (3)
- Increased a bit (4)
- Increased a lot (5)

**In this section, we want to understand more about the sexual experience of participants since social distancing measures due to the coronavirus began in March 2020.**
Has the number of your casual sex dates involving three or more people (e.g. threesome or group sex) changed since social distancing measures began in March due to the novel coronavirus?

- Reduced a lot (1)
- Reduced a bit (2)
- Stayed the same (3)
- Increased a bit (4)
- Increased a lot (5)

Has the number of your casual sex dates with one other person changed since social distancing measures began in March due to the novel coronavirus?

- Reduced a lot (1)
- Reduced a bit (2)
- Stayed the same (3)
- Increased a bit (4)
- Increased a lot (5)

Please indicate the (approximate) number (digits) of casual sex partners you have met for sex since social distancing began on the 24th of March.

________________________________________________________________

How long do you think you would be able to refrain from having casual sex because of the novel coronavirus?

- No time at all (1)
- Up to 1 week (2)
- Up to 2 weeks (3)
- Up to 4 weeks (4)
- Up to 3 months (5)
- Up to 6 months (6)
- More than 6 months (7)

What were the main reasons that you met casual sex partner/s since social distancing began in March? (please tick all that apply)

- I was horny (1)
- I was lonely and wanted some intimate physical contact (2)
- I was bored and wanted some distraction (3)
- I was afraid I might lose contact if we didn’t meet now (4)
- I was stressed and sex helps me relax (5)
- I was pressured into meeting sexual partner(s) (6)
- I needed to get away from my living place for a while (7)
- I needed to make money through sex work (8)
- I was in a cruising ground and it just happened (9)
- Any other reasons (please state) (10) ________________________________________________

When having casual sex dates, have you done anything to reduce your coronavirus transmission risk at any time? (Please tick all that apply)

- I've used hygiene techniques such as hand-washing or showering before and/or after sex. (1)
- I've had sex without mouth to mouth contact such as kissing. (2)
- I've had sex that involved physical distance (e.g. watching each other in the same room without touching) (3)
- I've changed where sex takes place. (4)
- I've used condoms for oral sex more frequently than I usually would. (5)
- I've used condoms for anal sex more frequently than I usually would. (6)
- I've avoided sex that might exposure me or my partner/s to faeces (poop) (e.g. not rimmed or been rimmed; been extra careful during or after fingering). (7)
- I've changed the type of sex I have during hook-ups (8)
- Other, please specify (9) ________________________________________________
- I have not changed any of my sexual practices (10)

**Please answer the next two questions about anxiety by ticking the box that best describes your experience over the last 6 weeks: 5.** How much of the time do you feel anxious about the coronavirus outbreak overall?

- At no time (1)
- Some of the time (2)
- Less than half of the time (3)
- More than half of the time (4)
- Most of the time (5)
- All of the time (6)

How much of the time do you feel anxious about catching coronavirus through casual sex?

- At no time (1)
- Some of the time (2)
- Less than half of the time (3)
- More than half of the time (4)
- Most of the time (5)
- All of the time (6)

**Below are some statements about feelings and thoughts. Please tick the box that best describes your experience of each over the last 6 weeks:**

|  | None of the time (1) | Rarely (2) | Some of the time (3) | Often (4) | All of the time (5) |
| --- | --- | --- | --- | --- | --- |
| I’ve been feeling optimistic about the future (1) |  |  |  |  |  |
| I’ve been feeling useful (2) |  |  |  |  |  |
| I’ve been feeling relaxed (3) |  |  |  |  |  |
| I’ve been dealing with problems well (4) |  |  |  |  |  |
| I’ve been thinking clearly (5) |  |  |  |  |  |
| I’ve been feeling close to other people (6) |  |  |  |  |  |
| I’ve been able to make up my own mind about things (7) |  |  |  |  |  |
